# Systematic survey reveals general applicability of "guilt-by-association" within gene coexpression networks

**DOI:** 10.1186/1471-2105-6-227

**Published:** 2005-09-14

**Authors:** Cecily J Wolfe, Isaac S Kohane, Atul J Butte

**Affiliations:** 1 Children's Hospital Informatics Program and Harvard MIT Division of Health Sciences and Technology, 300 Longwood Avenue, Boston, MA 02115, USA; 2 Hawaii Institute of Geophysics and Planetology, University of Hawaii at Manoa, 1680 East West Road, Honolulu, HI, 96822, USA; 3 Stanford Medical Informatics, Department of Medicine and Pediatrics, Stanford University School of Medicine, 251 Campus Drive, Room X-215, Stanford, CA 94305-5479, USA

## Abstract

**Background:**

Biological processes are carried out by coordinated modules of interacting molecules. As clustering methods demonstrate that genes with similar expression display increased likelihood of being associated with a common functional module, networks of coexpressed genes provide one framework for assigning gene function. This has informed the guilt-by-association (GBA) heuristic, widely invoked in functional genomics. Yet although the idea of GBA is accepted, the breadth of GBA applicability is uncertain.

**Results:**

We developed methods to systematically explore the breadth of GBA across a large and varied corpus of expression data to answer the following question: To what extent is the GBA heuristic broadly applicable to the transcriptome and conversely how broadly is GBA captured by *a priori *knowledge represented in the Gene Ontology (GO)? Our study provides an investigation of the functional organization of five coexpression networks using data from three mammalian organisms. Our method calculates a probabilistic score between each gene and each Gene Ontology category that reflects coexpression enrichment of a GO module. For each GO category we use Receiver Operating Curves to assess whether these probabilistic scores reflect GBA. This methodology applied to five different coexpression networks demonstrates that the signature of guilt-by-association is ubiquitous and reproducible and that the GBA heuristic is broadly applicable across the population of nine hundred Gene Ontology categories. We also demonstrate the existence of highly reproducible patterns of coexpression between some pairs of GO categories.

**Conclusion:**

We conclude that GBA has universal value and that transcriptional control may be more modular than previously realized. Our analyses also suggest that methodologies combining coexpression measurements across multiple genes in a biologically-defined module can aid in characterizing gene function or in characterizing whether pairs of functions operate together.

## Background

From the very start of the high-throughput microarray expression revolution it was understood [1,2] that guilt-by-association was a powerful heuristic to both explain why genes might have correlated expression in a set of experiments and infer what might be the function of a gene coexpressed with genes of better known function. As gene expression data have increased in numbers and quality, a variety of investigations have been leveraged from this GBA heuristic. Analyses of gene coexpression [3-7] have demonstrated that clusters with similar overall expression are often enriched for genes with similar functions, consistent with the hypothesis of modularly-behaving gene programs, where sets of genes are activated in concert to carry out functions.

GBA has also been exploited highly successfully by investigators who have used *a priori *determined modules or gene sets and assess if these sets have statistically significant overrepresentation in the genes changed in groups of arrays [8-15]. By exploiting the insight that subtle but coordinated changes in expression can be detected by combining measurements across multiple members of a functional module, these focused studies have successfully found specific modules that are important in diabetes [12], aging [13], and cancer [10,11,14,15], or assigned functions to previously uncharacterized genes in yeast [8,9]. These approaches essentially integrate two frameworks of viewing gene function [16], one framework reflected in module sets that are derived from prior biological knowledge and another framework from the characteristics of gene expression data.

These studies reflect two bidirectional uses of GBA: either using coexpression to define the members of functionally related sets or using sets to define function of coexpressed genes. That is, the first uses prior gene expression data and the second uses prior biological knowledge. We extend these approaches, taking the *a priori *framework of knowledge available in Gene Ontology (GO) [17] to systematically explore the breadth of GBA across a large and varied corpus of expression data to answer the following questions. 1) To what extent is the GBA heuristic broadly applicable to the transcriptome and GO? 2) In the GBA heuristic, how well does coexpression inform function and vice versa? 3) Which GO heuristics are the most interrelated as measured by a GBA metric?

The testbed for evaluating the extent and organization of GBA were five coexpression networks, constructed using 8341 microarrays representing a variety of tissue types and conditions. For each network we determine whether coordinated coexpression can be detected across multiple genes of each GO-defined module. Our approach is better suited than clustering to systematically examine GBA because it allows for pleiotropy: it does not assign genes to a single function or a single cluster but rather calculates a probabilistic score between each gene and each GO category. This approach better captures complex interrelationships [18], such as genes that code for proteins with multiple functions [19]. We discover that there is a ubiquitous signature of functional association in all of the coexpression networks in that the genes in a module often demonstrate higher-than-expected numbers of coexpressed genes belonging to that same module.

To further illustrate the breadth of GBA, we present the extent of which coexpression implicates members of three sets of genes that are usually thought of as belonging to a very specific biological context: skeletal development, neuropeptide receptor activity, and feeding behavior. We show that these Gene Ontology categories, as well as hundreds of other categories, are associated with coordinated expression patterns across the variety of tissue types and conditions in our data.

## Results and discussion

### Analysis of coexpression networks

We constructed five different coexpression networks (four single-species networks and one unified multi-species network), which are graphs where genes are nodes and the edges are represented by values reflecting the significance of coexpression between a pair of genes. We selected mammalian organisms for which extensive and diverse microarray data were available on four Affymetrix platforms in the Gene Expression Omnibus (GEO): *Homo sapiens *(HG-U95A and HG-U133A), *Mus musculus *(MG-U74A), and *Rattus norvegicus *(RG-U34A). Orthologs between these organisms were obtained from HomoloGene and from this information, 6624 "metagenes" (hereafter referred to as genes) were defined consisting of sets of orthologous genes across at least two different organisms on the chosen microarray platforms. The multi-species network integrates the 8341 microarrays from all four Affymetrix platforms into a unified coexpression network, using order statistics [6] to assign coexpression *P-*values (*P*_*c*_) between all possible pairs of genes. Previous work [6] suggested that by using the signal of evolutionary conservation in a multi-species coexpression network the effect of noise is reduced and the significance of functionally important gene pairs is enhanced, although this approach is only valid when homologous genes share functionality. The four single-species coexpression networks were calculated from Pearson correlation coefficients between genes, in each case using only data from one of the four Affymetrix platforms.

**Figure 1:**
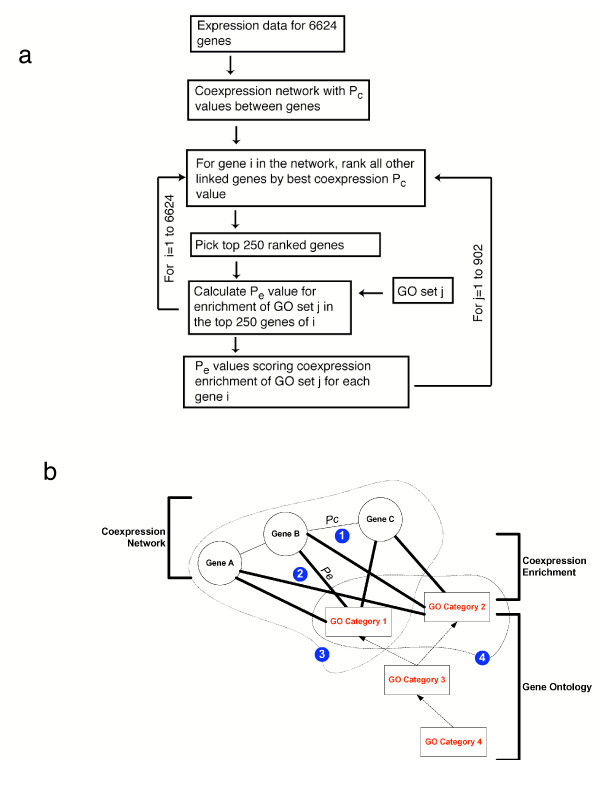
Schematic representation of the steps in our analyses. (a) Example flow chart of the different steps for calculating gene set coexpression enrichment *P*_*e *_values between each of the 6624 genes in the multi-species network and 902 GO sets. For each gene *m*_*i *_we use the hypergeometric distribution to calculate a coexpression enrichment *P*_*e*_-value (*P*_*e*_(*m*_*i*_, *g*_*j*_)) for whether GO set *g*_*j *_was significantly overrepresented in the top 250 genes with smallest *P*_*c*_-values to *m*_*i*_. (b) The four steps in our analyses. 1. A coexpression network is generated with *P*_*c *_values (multi-species network) or correlation coefficients (single-species network) scoring coexpression between gene pairs. 2. Coexpression enrichment *P*_*e *_values are calculated between each gene and each GO category, such as between GO category 1 and genes A, B, and C and between GO category 2 and genes A, B, and C. 3. A score reflecting GBA is calculated for each GO category (e.g., GO category 1). 4. The interrelationship between pairs of GO categories is quantified, such as that between GO category 1 and GO category 2, which are sibling categories in a Gene Ontology graph, sharing GO category 3 as a common parent.

For each network, we next construct a probabilistic score between each gene and each GO category that reflects the tendency for the genes in that GO set to be highly coexpressed with the selected gene. For each gene, a list of all other linked genes was ordered according to most significant coexpression *P*_*c*_-value (multi-species case) or highest correlation coefficient (single-species cases). For a given GO category, each gene in a coexpression network was analyzed using the hypergeometric distribution to determine if the GO set was overrepresented towards the top of the list of more highly correlated genes (Figure 1a). This process produces a gene set coexpression enrichment *P*-value (*P*_*e*_) between each of the genes and each of the GO sets. The *P*_*e*_-value between a particular gene and GO category is a probabilistic score for that pair, with lower (more significant) *P*_*e*_-values reflecting greater coexpression enrichment of that GO module. We demonstrate below how these *P*_*e*_-values have utility in identifying gene function, indicating the ubiquity of GBA across most GO categories, and how they quantify the interrelationships between GO categories (Figure 1b).

### Functional relevance of coexpression enrichment values

With a network of coexpression relations computed between pairs of genes, and networks of coexpression enrichment relations computed between all pairs of GO categories and genes, we evaluated how reliably coexpression enrichment *P*_*e*_-values for a GO category identify genes annotated with that function. Each GO category contains a set of specific *P*_*e*_-values to score relations to all genes. Taking one GO category at a time, we calculated the true and false positive rates for identifying genes annotated with that GO category at threshold *P*_*e*_-values throughout its range. We plotted these true and false positive rates on Receiver Operating Characteristic (ROC) curves [20] for each GO category. If there were a threshold *P*_*e*_-value below which all genes are annotated with the correct function, and above which no genes are annotated with the correct function, then the area under such an ideal ROC curve would be 1. An area of 0 would mean that identifying annotated genes using *P*_*e *_performs perfectly incorrectly, and an area of 0.5 indicates no overall identification efficiency using *P*_*e *_(performance equivalent to random chance). Thus the area under an ROC curve for each GO category is a metric for GBA, scoring how well coexpression enrichment *P*_*e*_-values perform as a "self-diagnostic" for the genes annotated to a category.

**Figure 2:**
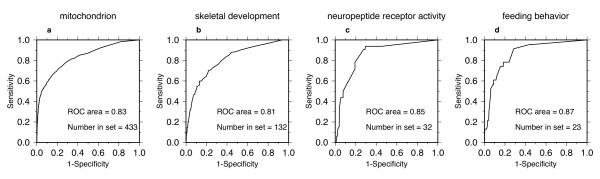
Examples from the multi-species network. (a-d) Self-diagnostic Receiver Operating Characteristic (ROC) curves for the GO categories shown above.

For the multi-species network, the ROC curves for the mitochondrion, skeletal development, neuropeptide receptor activity, and feeding behavior are all concave downward and plot above the diagonal (Figures 2a–d) with ROC areas greater than 0.5, indicating GBA for these GO categories. Skeletal development, neuropeptide receptor activity, and feeding behavior are usually thought of as belonging to a very specific biological context, yet genes in these categories are coexpressed across a wide range of samples from 8341 microarrays.

**Figure 3:**
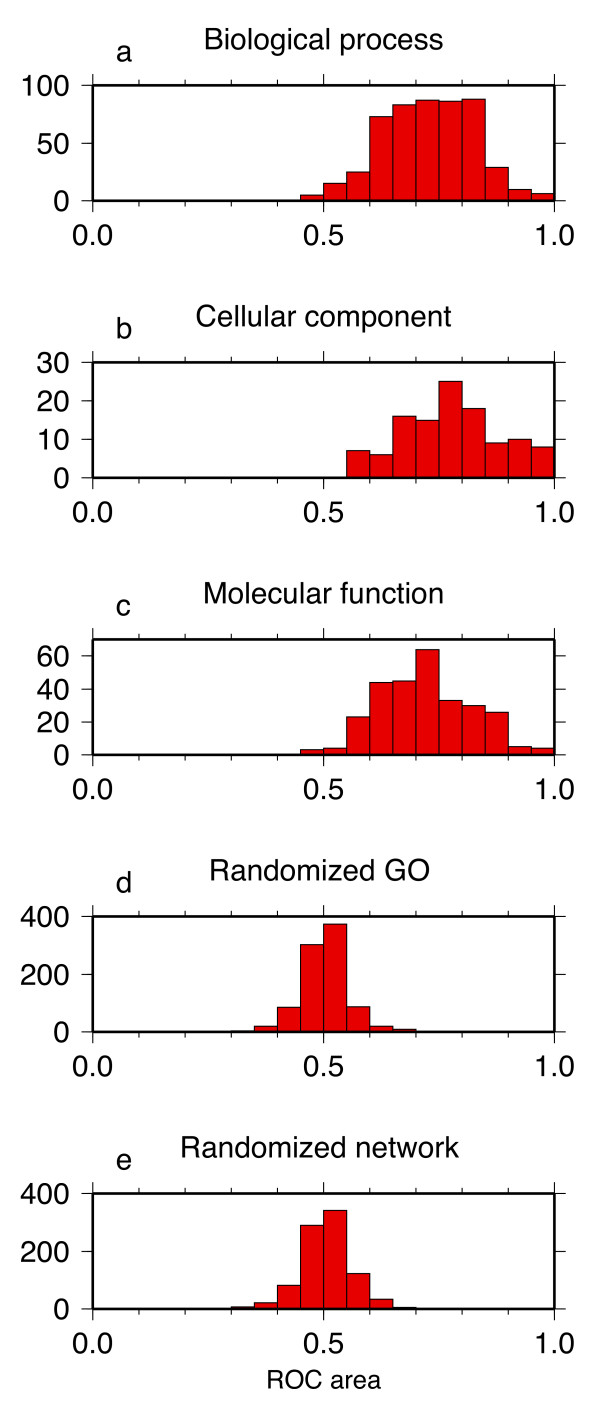
Histograms of self-diagnostic ROC areas for the multi-species network. (a) Histogram for biological process GO categories. (b) Histogram for cellular component GO categories. (c) Histogram for molecular function GO categories. (d) Histogram for randomized GO sets. (d) Histogram for a randomized multi-species coexpression network.

These patterns are typical of most GO categories. Self-diagnostic ROC areas for all of the GO categories in the multi-species network (see additional file 1: self-diagnostic ROC areas and 95% errors from the multi-species network), organized by the three domains of biological process, cellular component, and molecular function (Figures 3a–c), have distributions centered near 0.7, which is above the mean of 0.5 for the case where there would be no useful information in coexpression enrichment. This upward shift in the distributions indicates that for most GO categories, GBA is applicable and coexpression enrichment adds knowledge about gene function. This knowledge is not perfect: the ROC areas are all less than 1, and for many categories the large numbers of false positives at specific *P*_*e*_-value thresholds would limit the practical application of using this method to identify gene function. But nonetheless, a probabilistic signature of GBA is present. Equivalently, the members of a GO module as a whole tend to have more significant *P*_*e*_-values for that category than the non-members, because the ROC area also measures the probability that given randomly drawn pairs from two groups, one of members of a GO set and another of nonmembers, *P*_*e*_(member) <*P*_*e*_(nonmember) for coexpression enrichment of that set.

The results were tested by taking the multi-species coexpression network and applying the same analysis with randomized GO sets. The population of self-diagnostic ROC areas for the randomized GO sets is centered at 0.5 (Figure 3d). The case of a randomized network, with *P*_*c*_-values permuted between gene pairs, also yields a distribution that is centered at 0.5 (Figure 3e). Thus the upward shift of the true distributions is unlikely to occur by chance.

We tested whether the ROC areas were correlated with other factors (see additional file 2: supplementary methods), but found that the correlations were not strong, ranging between +/-0.2. We tested whether the type of evidence used to construct a GO set, given in the GO evidence codes, has any relation to the ROC areas; whether there was any correlation between the expression levels in a GO category and the ROC areas; whether there was a correlation between the ROC areas and the average number of GO annotations for the genes in each set.

### Interrelations between GO categories

To examine which GO categories are the most interrelated, we test whether coexpression enrichment for one GO set can be used to assign genes to a different GO category ("cross diagnostics"). These analyses score how well different GO modules tend to be coexpressed together, such as whether coexpression enrichment for the mitochondrion module is a characteristic of the oxidative phosphorylation module (the multi-species cross-diagnostic ROC area is 0.94 for this case). In one sense, these scores indicate the strength of coexpression links in a network where the graph nodes are GO categories, rather than genes. However, a complication is that pairs of gene sets may significantly overlap in their annotated genes. Therefore, for the multi-species network we present the systematics between pairs of GO categories that are together on the same graph, where GO relationships are defined and provide additional context for interpreting the results. Gene Ontology organizes biological processes, molecular functions, and cellular components separately on three directed acyclic graphs. A parent GO category has a set of more specific children (from those GO categories just one step below a parent on a graph) and more specific descendents (from all GO categories in the entire subgraph below a parent). To test the results, we apply the same analysis with randomized GO sets that are constructed in a manner that mimics the GO mappings.

**Figure 4:**
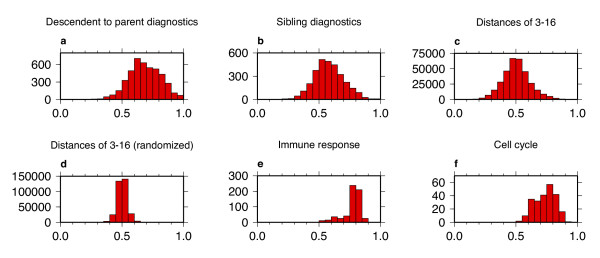
Histograms of cross-diagnostic ROC areas for the multi-species network. (a) Histogram of ROC areas for whether descendent *P*_*e*_-values are diagnostic of parent sets. (b) Histogram of ROC areas for cross pairing of sibling categories. (c) Histogram of ROC areas for all cross pairings of categories (excluding parent-descendent pairs) with distances of 3–16 in a GO graph. GO organizes categories as nodes on a graph and calculates the distance between category pairs on the same graph as the minimum number of arcs needed to traverse from one category node to another on the graph. For example, a parent and its child are separated by a distance of one and siblings are separated by distances of two. (d) Histogram of ROC areas for all cross pairings of categories (excluding parent-descendent pairs) with distances of 3–16 in a GO graph, created using randomized GO sets. (e) Histogram of cross-diagnostic ROC areas between GO category pairs (excluding parent-descendent pairs) in the subgraph below immune response. (f) Histogram of cross-diagnostic ROC areas between GO category pairs (excluding parent-descendent pairs) in the subgraph below cell cycle.

For cross-diagnostic tests of whether *P*_*e*_-values of descendent GO categories can correctly identify genes in parent GO categories (Figure 4a), we find that the distribution is shifted above 0.5 (mean = 0.67). However, descendent sets are subsets of parent sets, so it is consistent that this distribution is similar to the patterns in the self-diagnostic ROC areas. We next examine GO categories that are siblings (children of a common parent), since GO children split a parent into distinct and more specialized categories. For sibling pairs (Figure 4b), the shift above 0.5 is less (mean = 0.57). Yet the populations of ROC areas across sibling pairs and across descendent-parent pairs remain more diagnostic than the population across more distantly-related pairs (Figure 4c), which is centered at the expected mean of 0.5 for the case of no interrelation on average.

However, the distribution (Figure 4c) does display longer tails than for randomized GO sets (Figure 4d), indicating how there is a nonrandom tendency for some of these modules to either be highly coexpressed together (high areas) or not highly coexpressed together (low areas). (Note that increasing the scale in Figure 4d does not reveal any additional detail in the tails of the distribution.) In addition, some subgraphs of GO show uniformly high cross diagnostics, such as the subgraphs under immune response and cell cycle (Figure 4e–f), where there is a signal that modules from the different sub-categories are often coexpressed together in the types of tissues in our analysis.

Of the 812,702 possible cross diagnostic GO pairings, only a small percentage are related by coexpression (e.g., 5% have ROC areas greater than 0.7). As shown in the above analyses, at least some of the positive relationships are consistent with the known biology reflected the Gene Ontology hierarchy.

### Reproducibility across different microarray platforms

**Figure 5:**
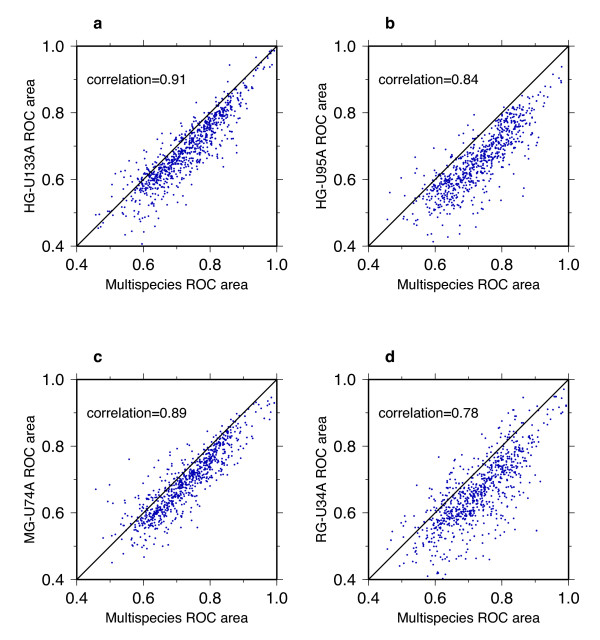
Plots of self-diagnostic ROC areas from the multi-species network (x-axis) versus ROC areas from a single-species network (y-axis) for each GO category. Each panel examines one of the single-species networks, created using microarrays from the following Affymetrix platforms: HG-U133A (human), HG-U95A (human), MG-U74A (mouse), and RG-U34A (rat). Correlation coefficients are noted in the upper left corner of the plots.

The patterns found in the multi-species network are highly reproducible in the single-species networks for each of the 4 different microarray platforms. Self-diagnostic ROC areas derived from single-species networks are strongly correlated with the values derived from the multi-species network with correlation coefficients ranging from 0.8 to 0.9 (Figure 5). However, the ROC areas from single-species networks typically are lower than areas from the multi-species network, plotting below a diagonal straight line of slope one and zero intercept. This shift likely arises because the multi-species coexpression network reduces the effects of noise and enhances the ability of the network to link with more significance gene pairs involved in common function [6]. But for our analysis this enhancement is only minor, illustrating how coexpression from a single organism already captures the signal of GBA. The cross-diagnostic ROC areas are also strongly correlated in the five different networks, with correlation coefficients between single-species and multi-species cross-diagnostic ROC areas ranging from 0.7 to 0.9 (data not shown). The interrelatedness between pairs of GO modules is therefore also reproducible across the different datasets on the different platforms.

The observed scores appear to reflect the behavior of the transcriptome rather than being dominated by the mix of samples in each of the networks or the choice of microarray platform. Though the GEO data in our study originated from many laboratories with inhomogeneous protocols, our analyses demonstrate how the extent of GBA for each GO module and the interrelatedness between GO module pairs nonetheless have high reproducibility in the networks. The reproducibility between the entire multi-species and single-species networks is lower than the reproducibility of ROC areas (see additional file 2: supplementary methods), demonstrating how a functionally-based analysis enhances the similarity of the signals between different networks. Our results are consistent with a recent study of expression variability across different platforms and laboratories [21] that found highest reproducibility when the analysis was based on biological themes defined by GO.

## Conclusion

Our study provides an investigation of the functional organization of five coexpression networks using data from three mammalian organisms. This method integrates information from two different frameworks of viewing gene function [16], one framework essentially from the manual and subjective curation of evidence in the literature into the Gene Ontology hierarchy and another framework from a probabilistic analysis of expression datasets. Across all five networks, we find a signature that coexpression enrichment predicts coannotation across GO categories, and thus the guilt-by-association heuristic is broadly applicable. Although for gene pairs within a specified GO set the coexpression value may only be weak, by combining coexpression measurements across multiple genes in the module, there is a systematic and reproducible signature of functional association. Because the genes in a particular module demonstrate higher-than-expected numbers of coexpressed genes belonging to that same module, the values for gene set coexpression enrichment tend to be predictive of gene function.

It was unexpected that a simple test based on coexpression would have value in assigning genes to so many different types of GO categories. While some GO annotations may themselves have been defined on the basis of expression, there are also many GO annotations that did not necessarily employ expression results, such as the annotations in the cellular component domain, where the population of ROC areas still displays better-than-random ability to correctly identify the genes annotated to GO categories. That some GO categories score better than others likely reflects the characteristics of underlying biological behavior, as the scores of GO categories are reproducible across all of the coexpression networks. This study demonstrates how using coexpression enrichment to assign a probabilistic score between genes and functions can add information about gene function. We note that an analogous data mining approach to ours was previously applied by Lamb *et al*. [11] to discover that C/EBPβ was a mechanism of cyclin D1 action, using a single module gene set of cyclin D1 target genes. Our more comprehensive study of 902 GO module gene sets suggests this type of approach should also be successful for other biological systems. Our results are in agreement with a recent study [22] that used a support vector machines method on mouse coexpression data and found that genes in many GO biological process categories could be identified as being in those categories. Our results disagree with low degree of GBA found by Clare and King [23], who clustered yeast microarray data and found the clusters did not in generally agree with functional annotation classes. One explanation for this disagreement may be that the use of clustering by Clare and King [23] does not reveal the more subtle signal of GBA that we discover using gene set coexpression enrichment. Another difference may be that our larger and more comprehensive dataset (8341 Affymetrix mammalian microarrays) is better suited to identify GBA.

Our strategy demonstrates that the functions of a cell operate on an exquisitely coordinated level and that the modular character of cell biology [24] is evident across the biologically variable microarray data in our analysis. Within the large scope of the considered GEO samples and GO categories, we find that the guilt-by-association identification of gene function on the basis of expression has universal value. This result provides optimism that high-throughput measurements of gene expression and community-based gene annotation efforts will continue to demonstrate synergy in the collective investigations of cellular physiology and understanding of human diseases.

## Methods

### Assignment of metagenes

Genes from one organism were associated with their orthologous counterparts in other organisms using HomoloGene (downloaded on June 22, 2004). 6624 "metagenes "were defined as sets of orthologs across at least two organisms with available microarray probes, using cases where no more than one gene was found for each organism. Microarray probes for orthologs were assigned into metagene probe groups. Because some genes have multiple probes on an array, for each of the 6624 metagenes, we considered all combinations of probes across the microarray platforms (see additional file 2: supplemental methods).

### Generation of coexpression networks

Microarray data consisted of 8341 arrays from 4 different platforms downloaded from NCBI Gene Expression Omnibus. 2179 arrays were Affymetrix HG-U95A (version 2), 2438 arrays were Affymetrix HG-U133A, 2216 arrays were Affymetrix MG-U74A (version 2), and 1508 arrays were Affymetrix RG-U34A. We normalized expression data on each array by converting values to rank percentile. For the multi-species network, for each probe group we computed Pearson correlation coefficients between other probes on a platform and then ranked these other probes according to their correlations. For each distinct pair of metagene probe groups, a probabilistic method based on order statistics was used to evaluate the probability of observing the ranks by chance (see additional file 2: supplemental methods). This generates coexpression *P-*values (*P*_*c*_(*m*_*i*_*, m*_*j*_)) between pairs of metagenes. A unique *P*_*c*_-value between metagene pairs is selected based on lowest *P*_*c*_-value obtained from all of the analyzed probe groups, with the philosophy that when coexpression is present, more significant *P*_*c*_-values will be associated with more accurate probes. Single-species coexpression networks for each of the four different platforms were calculated from Pearson correlation coefficients between gene pairs, limited to those genes also analyzed in the multi-species network and selecting the highest correlation coefficient obtained for the cases where multiple probes are available for gene pairs.

### GO gene sets

For each network, gene sets were compiled for 902 GO categories with at least 20 genes in the multi-species network. The graph relationships were obtained from the Gene Ontology MySQL database, downloaded on September 24, 2004. The annotations of genes to GO categories were taken from LocusLink, downloaded on September 27, 2004. Gene Ontology organizes biological processes, molecular functions, and cellular components separately on three directed acyclic graphs, with more general parent categories having subgraphs of more specific descendent categories. The GO true path rule is that annotation to a category implies annotation to all parents and gene products are conventionally annotated just to the most specific levels of the ontology. We associate a gene to a GO set if it is annotated with that GO category in human, mouse, or rat or if it is annotated with a descendent of that GO category. See additional file 2: supplemental methods, for the construction of the randomized GO sets.

### Statistical significance of coexpression enrichment of a GO set

For each gene *m*_*i*_, all other linked genes are ranked by the most significant value obtained for coexpression. We use the hypergeometric distribution to calculate a coexpression enrichment *P-*value (*P*_*e*_(*m*_*i*_, *g*_*j*_)) for whether GO set *g*_*j *_was significantly overrepresented in the top 250 genes with most significant coexpression values to *m*_*i *_(Figure 1). Similar results were obtained for cases where the number of top ranked genes selected was different (decreased to 100 or increased to 500) or where an enrichment score was based on a normalized Kolmogorov-Smirnov statistic [12].

### Receiver Operating Characteristic (ROC) curves

A self-diagnostic ROC curve tests whether the *P*_*e*_(*m*_*i*_, *g*_*j*_)-values for GO set *g*_*j *_can distinguish genes associated to *g*_*j*_. An ROC curve is constructed for a range of closely spaced *P*_*e*_(*m*_*i*_, *g*_*j*_)-value cutoffs. At a given cutoff, the true-positive rate (sensitivity) is calculated as the number of genes associated to GO set *g*_*j *_with *P*_*e*_(*m*_*i*_, *g*_*j*_)-values below the cutoff divided by the total number associated to *g*_*j*_; the false-positive rate (1-specificity) is calculated as the number of genes not associated to a GO set *g*_*j *_with *P*_*e*_(*m*_*i*_, *g*_*j*_)-values below the cutoff divided by the total number not associated to a GO set. The area is estimated by trapezoidal integration and 95% confidence intervals are also calculated [20]. Cross-diagnostic ROC areas are calculated as above, except we test whether the *P*_*e*_(*m*_*i*_, *g*_*j*_)-values for *g*_*j *_can distinguish genes associated to a different GO set *g*_*k*_.

## Authors' contributions

This study was conceived by AJB and CJW. CJW carried out the analyses, with advice and guidance from AJB and ISK. All authors fully participated in the interpretation of results and the writing of the manuscript.

## Supplementary Material

Additional File 1 Self-diagnostic ROC areas and 95% errors from the multi-species network. This file lists ROC areas for the 902 GO categories. Click here for file

Additional File 2 Supplemental Methods. This file provides supplemental information on the methods used in the analyses. Click here for file
