# Supplementary material for: Systematic survey reveals general applicability of "guilt-by-association" within gene coexpression networks
Source: BMC Bioinformatics. 2005 Sep 14;6:227. doi: 10.1186/1471-2105-6-227 (PMC1239911; doi:10.1186/1471-2105-6-227)
Supplement: Additional File 2 — Supplemental Methods. This file provides supplemental information on the methods used in the analyses. [file 1471-2105-6-227-S2.doc]

# Additional File 2: Supplemental Methods

# Examples of probe groups

Microarray probes for orthologs were assigned into metagene probe groups. Because some genes have multiple probes on an array, for each of the 6624 metagenes, we considered all combinations of probes across the 4 chosen microarray platforms (additional file 1), yielding 24,087 metagene probe groups. To illustrate our procedure, an example table of probe groups is shown below. Each different probe group is assigned a unique number (first column). The metagene ID (sixth column) corresponds to either the human NCBI GeneID or, in cases where no human ortholog is found, the mouse NCBI GeneID. Metagene 38 (ACAT1) and metagene 732 (C8B) are examples where multiple probe groups map to a common metageneID. Metagene 215 (ABCD1) will not be linked with either metagene 732 or metagene 38, because correlations involving probes from at least two different species cannot be calculated. The 6624 metagenes in the network have 3 probes on average assigned to them.

Group # HG-U95A MG-U74A RG-U34A HG-U133A Metagene ID

| 1 | 37532_at | 92581_at | J02791_at | 202502_at | 34 |
| --- | --- | --- | --- | --- | --- |
| 2 | 39678_at |  | D00512_at | 205412_at | 38 |
| 3 | 39678_at |  | D00512_g_at | 205412_at | 38 |
| 4 | 39678_at |  | D13921_s_at | 205412_at | 38 |
| 5 |  | 98969_at |  | 205142_x_at | 215 |
| 6 | 36304_at |  | U20194_at | 206979_at | 732 |
| 7 | 36304_at |  | U20194_g_at | 206979_at | 732 |

# Construction of the multiple species coexpression network

For every linked pair of metagenes, the probability of observing their gene-gene correlations by chance was computed by order statistics. Let *g_ij_* be a gene belonging to probe group *i* on platform *j*. For a specific *i* and *j*, we rank all other probes on platform *j* to relative to *g_ij_* based on their Pearson correlation coefficients, in order of smallest to largest, using only probes that map to different metagenes. Let *N_1_*, *N_2_*, *N_3_*, and *N_4_* be the number of correlations to *g_i1_*, *g_i2_*, *g_i3_*, and *g_i4_*. To obtain normalized ranks, we divide the ranks by the number of correlations, so for example, for *g_i1_* the normalized ranks on the first platform will range from 1/*N_1_* to 1, with more negative correlations corresponding to smaller normalized ranks and more positive correlations corresponding to larger normalized ranks. To evaluate the significance of the correlations between two probe group pairs *P(i, i’)*, we calculate the normalized ranks *r_1_*,…, *r_n_* of *i’* relative to *i* and compute the probability of observing them by chance. If we assume that the normalized ranks are drawn independently and uniformly, a directed *P* value can be calculated from the joint cumulative distribution of an *n*-dimensional order statistic.

*P(r_1_,…, r_n_)*=*n!  ∫_0_^r1^∫_s1_^r2^…∫_sn-1_^rn^ds_1_…ds_n_*

In our case, *n*=2 to 4, depending on how many platforms are available between the two probe groups *i* and *i’*. *r_1_*,…, *r_n_* is a particular configuration of normalized ranks across the *n* different platforms, ordered from smallest to largest. As an illustrative example, take the case of rolling 4 dice and obtaining the result (1, 2, 3, 4), ordered from smallest to largest. When the values are normalized by 6, the *P*-value from above integral will calculate the probability of rolling 4 dice and an obtaining an ordered result (*X_1_*<1/6, *X_2_* <2/6, *X_3_* < 3/6, *X_4_*<4/6). This example evaluates significance in the direction of small ranks, which for our ordering of correlation coefficients would evaluate significance in the direction of negative correlation. For coexpression *P*-values, we calculate significance in the direction of positive correlation by using the symmetry in order statistic *P*-values: e.g., *P*(*X_1_*>3/6, *X_2_* >4/6, *X_3_* > 5/6, *X_4_*>6/6)*=P*(*X_1_*<1/6, *X_2_* <2/6, *X_3_* < 3/6, *X_4_*<4/6). After all analyses have been performed, we construct a network where a unique coexpression value *P_c_* between a given pair of metagenes (*m_i_*, *m_j_*) is assigned as the most significant *P* value for coexpression obtained for that pair.

**Construction of randomized GO sets**

To construct GO randomized sets, we counted up the number of genes annotated to each GO category in LocusLink and then randomly assigned the same number of genes to that GO category. Each gene for the randomized set was then associated to a GO category using the GO graph relationships. This process generates randomized GO associations while mimicking the effects of the GO graph relationships. The randomized sets test the case where no prior information is used in the annotations. We find that the overall diagnostic ability is also poor in tests where all of the gene annotations to GO categories from LocusLink are instead randomly permuted, although this second case retains the prior information regarding frequency of GO annotations for each metagene in LocusLink.

# Tests of whether ROC areas are correlated with GO evidence codes

We find that the ROC scores are not well correlated with the proportion of GO evidence codes in a gene set as listed in the annotation file obtained through LocusLink. For each GO evidence code, we score the proportion of genes in a GO set that have been annotated with that evidence code. The proportion ranges from 0 (no genes in a set used that evidence code) to 1 (all genes in that set used that evidence code). We then calculate the correlation coefficient between ROC areas for GO sets and the evidence code proportion for GO sets. None of the correlations exceeds +/-0.2, indicating that the ROC areas are not well correlated with proportion of GO evidence codes for the gene sets.

IC (inferred by curator): correlation=0.07

IDA (inferred from direct assay): correlation=-0.19

IEA (inferred from electronic annotation): correlation=0.16

IEP (inferred from expression pattern): correlation=-0.03

IGI (inferred from genetic interaction): correlation=-0.06

IMP (inferred from mutant phenotype): correlation=-0.06

IPI (inferred from physical interaction): correlation=-0.12

ISS (inferred from sequence or structural similarity): correlation=0.08

NAS (non-traceable author statement): correlation=-0.06

ND (no biological data available): correlation=-0.06

NR (not recorded): correlation=0.13

RCA (inferred from reviewed computational analysis) No annotations have

this evidence

TAS (traceable author statement): correlation=0.05

# Tests of whether ROC areas are correlated with expression levels

For the single-species networks, for each GO category we test whether there is any correlation between the average of genes expression levels and the ROC areas, to examine whether the low ROC areas are due to genes with low expression levels and poorer signal-to-noise ratios. We find no strong correlation between the average expression levels and ROC areas.

HG-U95A: correlation=0.08

HG-U133A: correlation=0.15

MG-U74A: correlation=-0.05

RG-U34A: correlation=0.08

Test of whether ROC areas are correlated with the average number of GO annotations for the genes in each set

We examined the correlation between ROC area versus the average number of GO annotations for the genes in each set and found a weak negative correlation=-0.19.

To perform the analysis, we counted up how many GO annotations each gene has, only counting annotations at the most specific levels of the graphs, limited to the 902 GO categories (e.g., when a gene is counted to hit a GO category, it means the gene is not annotated in the GO graph below that category and the annotations to all of the GO parents/grandparents etc. above in the graph above are not counted. However, a gene's annotations to two sibling GO categories would both be counted). Then for each GO category, we calculate the average number of GO annotations for all genes in the GO set. For most categories, genes are on average annotated to about 8 other specific GO categories (at the most specific levels of the graphs, considering only those 902 GO categories with at least 20 genes in the multispecies network).

**Multispecies versus single species coexpression network reproducibility**

The correlations between self-diagnostic ROC areas in the multispecies versus single species networks range from 0.8-0.9 (Figure 5). The cross diagnostic ROC correlations between multi-species and single-species networks range from 0.7-0.9. We here examine whether these functional correlations could simply reflect the correlation between the entire coexpression networks.

Because our methodology is rank based (Figure 1), we examine whether the gene ranks the multispecies network are correlated with the gene ranks in the single species networks. For each gene, a list of all other linked genes was ordered according to most significant coexpression *P_c_*-value (multi-species case) or highest correlation coefficient (single-species cases). The ranks of common genes (normalized to linearly range between 0 and 1) were then paired across both types of networks and the correlation coefficient using all possible pairs was calculated. The results are listed below.

Multi-species vs. Affymetrix MG-U74A network correlation coefficient of ranks: 0.54

Multi-species vs. Affymetrix RG-U34A network correlation coefficient of ranks: 0.45

Multi-species vs. Affymetrix HG-U95A network correlation coefficient of ranks: 0.34

Multi-species vs. Affymetrix HG-U133A network correlation coefficient of ranks: 0.38

The reproducibility between entire networks is lower than the reproducibility between ROC areas, demonstrating how the functionally-based signals are more similar than the ranks of raw coexpression signals.
